# Involvement of TRPM2 and TRPM8 in temperature-dependent masking behavior

**DOI:** 10.1038/s41598-019-40067-x

**Published:** 2019-03-06

**Authors:** Wataru Ota, Yusuke Nakane, Makiko Kashio, Yoshiro Suzuki, Kazuhiro Nakamura, Yasuo Mori, Makoto Tominaga, Takashi Yoshimura

**Affiliations:** 1 Institute of Transformative Bio-Molecules (WPI-ITbM), Nagoya University, Furo-cho, Chikusa-ku, Nagoya, 464-8601 Japan; 2 Laboratory of Animal Integrative Physiology, Graduate School of Bioagricultural Sciences, Nagoya University, Furo-cho, Chikusa-ku, Nagoya, 464-8601 Japan; 3 Avian Bioscience Research Center, Graduate School of Bioagricultural Sciences, Nagoya University, Furo-cho, Chikusa-ku, Nagoya, 464-8601 Japan; 4 Department of Physiology, Aichi Medical University, 1-1 Yazakokarimata, Nagakute, 480-1195 Japan; 5 Division of Cell Signaling, National Institute for Physiological Sciences, National Institutes of Natural Sciences, 5-1 Higashiyama, Myodaiji, Okazaki, 444-8787 Japan; 6 Thermal Biology Group, Exploratory Research Center on Life and Living Systems, National Institutes of Natural Sciences, 5-1 Higashiyama, Myodaiji, Okazaki, 444-8787 Japan; 7 Department of Integrative Physiology, Nagoya University Graduate School of Medicine, 65 Tsurumai-cho, Showa-ku, Nagoya, 466-8550 Japan; 8 Department of Synthetic Chemistry and Biological Chemistry, Graduate School of Engineering, Kyoto University, Nishikyo-ku, Kyoto, 615-8510 Japan; 9 Division of Seasonal Biology, National Institute for Basic Biology, National Institutes of Natural Sciences, 38 Nishigonaka, Myodaiji, Okazaki, 444-8585 Japan

## Abstract

Masking is a direct behavioral response to environmental changes and plays an important role in the temporal distribution of activity. However, the mechanisms responsible for masking remain unclear. Here we identify thermosensors and a possible neural circuit regulating temperature-dependent masking behavior in mice. Analysis of mice lacking thermosensitive transient receptor potential (TRP) channels (*Trpv1/3/4* and *Trpm2/8*) reveals that temperature-dependent masking is impaired in *Trpm2*- and *Trpm8*-null mice. Several brain regions are activated during temperature-dependent masking, including the preoptic area (POA), known as the thermoregulatory center, the suprachiasmatic nucleus (SCN), which is the primary circadian pacemaker, the paraventricular nucleus of the thalamus (PVT), and the nucleus accumbens (NAc). The POA, SCN, PVT are interconnected, and the PVT sends dense projections to the NAc, a key brain region involved in wheel-running activity. Partial chemical lesion of the PVT attenuates masking, suggesting the involvement of the PVT in temperature-dependent masking behavior.

## Introduction

The circadian clock, a 24-hour endogenous biological timer, is highly conserved in virtually all living organisms. This clock regulates various physiological and behavioral processes, such as sleep–wake cycles and metabolism. Entrainment and masking are two independent processes that determine whether animals exhibit diurnal or nocturnal behavior^1,2^. Entrainment refers to synchronization of the circadian clock to environmental cycles, whereas masking is a direct response to environmental signals with a change in activity. Interplay between entrainment and masking results in the distribution of locomotor activity to a specific time of day, known as the temporal niche. Entrainment of circadian rhythms is mediated by light information received by rods, cones, and melanopsin (OPN4)-expressing retinal ganglion cells. This information is then conveyed to the suprachiasmatic nucleus (SCN), the central circadian pacemaker located in the hypothalamus, directly via the retinohypothalamic tract (RHT) and indirectly from the intergeniculate leaflet (IGL) via the geniculo-hypothalamic tract (GHT)^3–6^. Thus, the photoreceptors and neural circuits involved in photoentrainment are well established. In marked contrast, the mechanism(s) responsible for masking behavior remain unclear.

Non-mammalian vertebrates perceive light information directly within the brain via deep brain photoreceptors^7^. In previous studies, we found that OPN5-positive cerebrospinal fluid (CSF)-contacting neurons within the hypothalamus are among the deep brain photoreceptors that regulate seasonal reproduction in birds^8,9^. Interestingly, light also penetrates the brain of some mammalian species^10^, and OPN5 is expressed in the mouse and human brain^11,12^. However, the physiological function of OPN5 within the mammalian brain is unknown.

In an effort to understand the physiological function of OPN5 in the mouse brain and test for the possible existence of extra-ocular photoreception in mice, we serendipitously observed suppression of locomotor activity in blinded mice. Further experiments demonstrated that the phenomenon we observed was, in fact, temperature-dependent masking behavior. We went on to identify two thermosensors (TRPM2 and TRPM8), which regulate this adaptive behavior by analyzing all available transient receptor potential (TRP) channel–null mice (*Trpv1/3/4* and *Trpm2/8*). Moreover, we propose a possible neural circuit that mediates this behavior based on expression analysis of the neuronal activation marker *Fos* and chemical lesions in the brain. In particular, the paraventricular nucleus of the thalamus (PVT) may be an important interface that regulates temperature-dependent masking behavior.

## Results

### Blinded mice exhibit masking behavior during UVA light exposure

**Figure 1:**
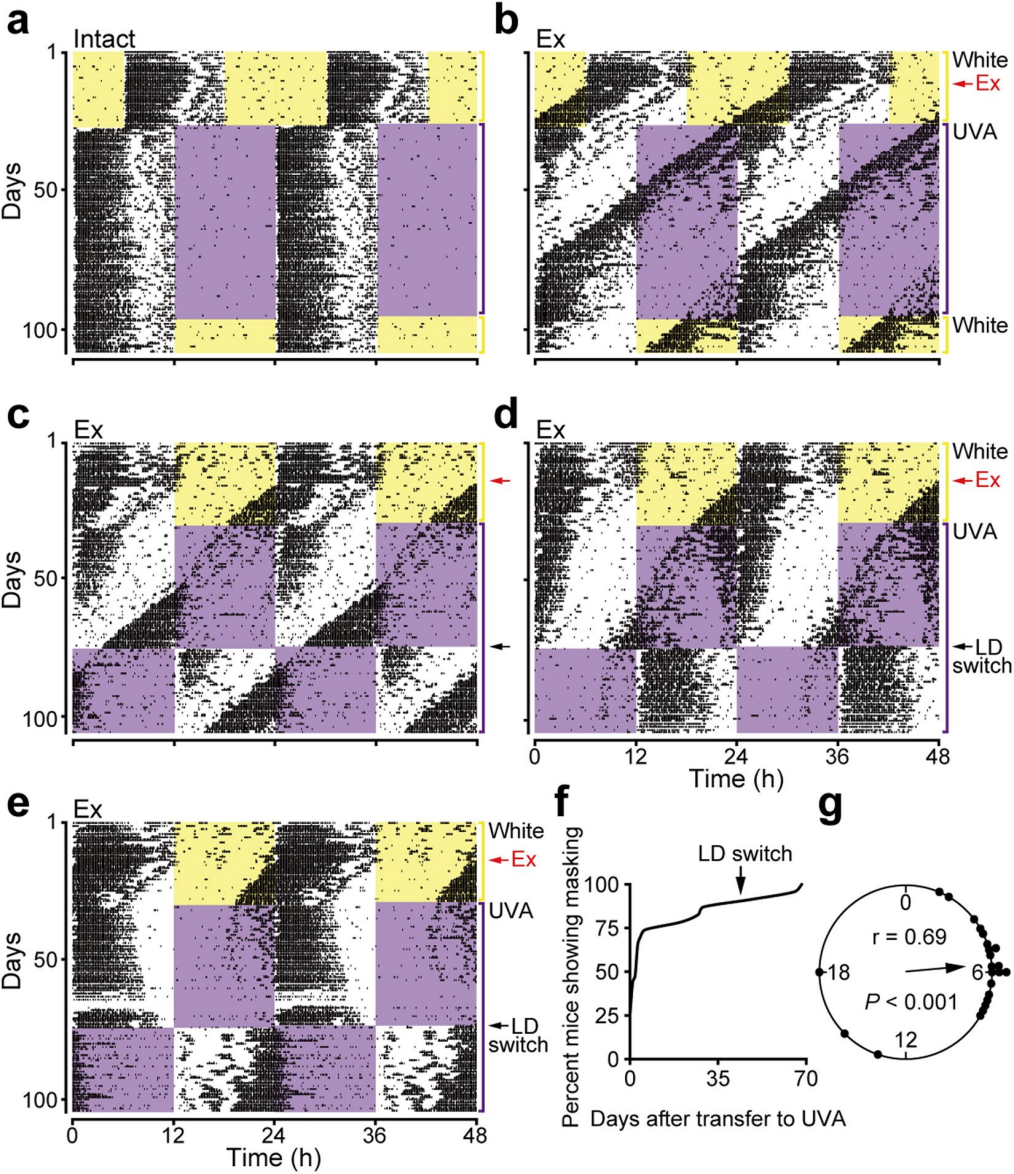
Blinded C57BL/6 J mice exhibit masking behaviors during UVA light exposure. (**a**,**b**) Representative actograms of intact mice (**a**) and enucleated (Ex) blinded mice. (**b**) The 12-h light periods are shown as colored background (yellow: white light, purple: UVA light [λ_p_ = 365 nm]). Ex group mice were bilaterally enucleated on day 14 (Red arrows). (**c**–**e**) Representative actograms of blinded mice in the validation experiment. (**f**) Percentage of animals exhibiting the negative masking behavior in UVA light phase. (**g**) Rayleigh plot of the phases for the beginning of negative masking behavior. Individual data are plotted on the circle (n = 23). The direction of the arrow indicates the mean phase vector, and the length represents the strength of the phase clustering (*r* value). The *p* value is based on the Rayleigh test.

Since mouse and human OPN5 exhibit absorption maxima in the ultraviolet A (UVA) range (360–380 nm)^12,13^, we first examined the effect of UVA light on wheel-running activity of blinded C57BL/6 J mice to investigate whether mice have the capacity for extra-ocular photoreception. As expected, intact mice entrained to LD cycles of both white light and UVA light (Fig. 1a), whereas blinding caused free-running rhythms under both of these lighting conditions (Fig. 1b) (n = 5). These results are consistent with previous reports showing that eyes are the only photoreceptive organ in mammals^14,15^. Surprisingly, however, we observed decreased locomotor activity, an example of negative masking behavior^1,2^, in blinded mice during exposure to UVA light (Fig. 1b). Although negative masking was observed in all blinded mice tested, the free-running period and extent of masking varied among individuals. Therefore, to further characterize this variability, we analyzed wheel-running activity in additional blinded mice (n = 23). Although the typical free-running period of C57BL/6 J mice is a bit shorter than 24 hours, blinded mice exhibited a broader range and decreased stability in free-running period (Fig. 1c–e). These variations are likely due to the absence of retinal circadian oscillators, which are normally coupled to and interact continuously with the circadian pacemaker in the SCN^16^. About 70% of blinded mice exhibited masking behaviors immediately after transfer to a UVA-LD cycle (Fig. 1f), whereas the rest of the animals developed masking behaviors gradually. When we examined the onset of masking, we noticed that blinded mice exhibited masking behavior when the onset of UVA light coincided with the middle of the subjective day (i.e., circadian time ~6; circadian time 12 is defined as the time of activity onset in nocturnal animals under constant conditions) (Fig. 1g). Phase-specific negative masking effects in mice have also been reported by Hoffmann^17^. Thus, the variation in the onset of masking behavior appears to depend on the large variation in free-running period caused by blinding. In any event, all animals exhibited masking behavior by 70 days after transfer to a UVA-LD cycle (Fig. 1f).

### Temperature stimulus causes negative masking behavior in blinded mice

**Figure 2:**
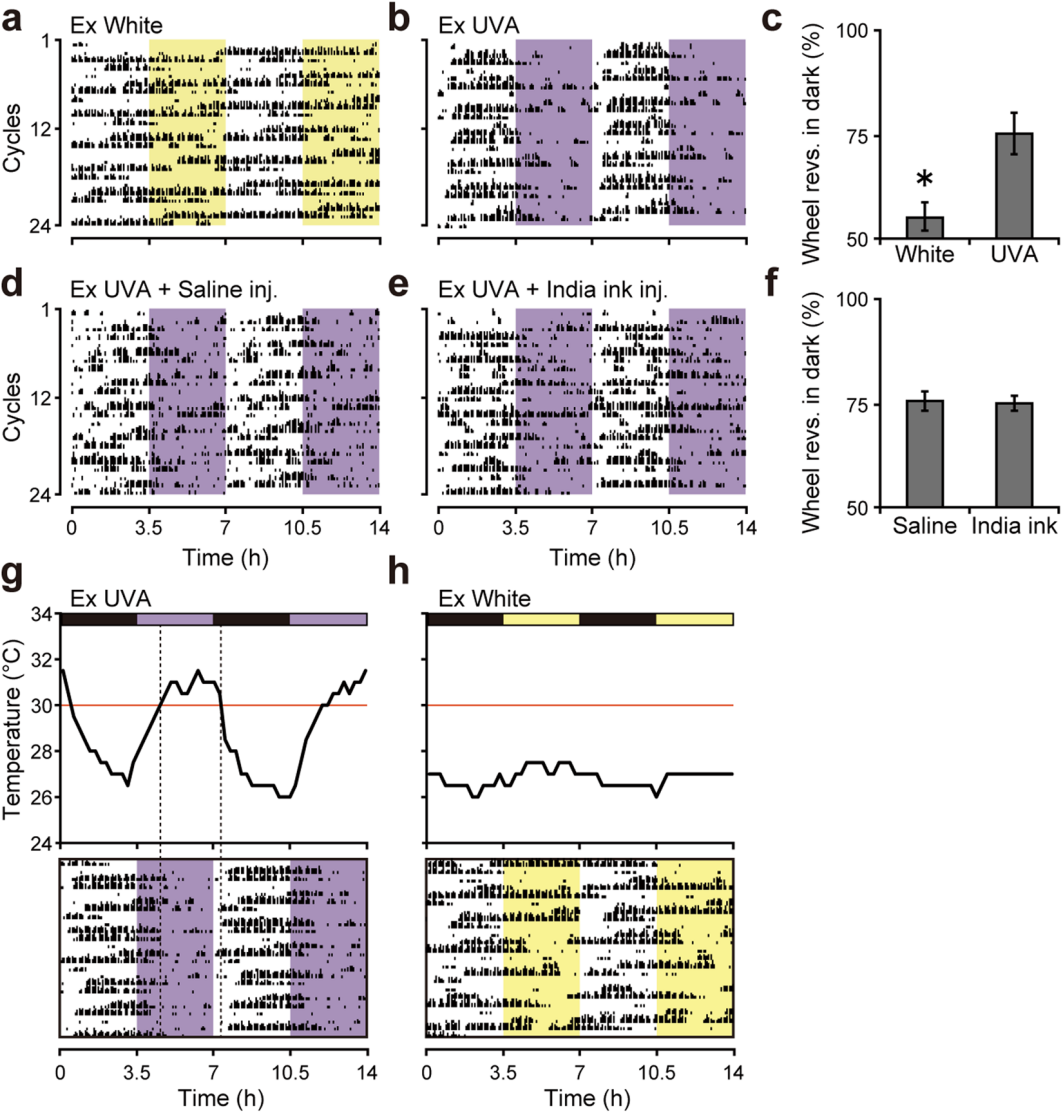
Temperature stimulus generated by UVA light causes negative masking behaviors in blinded mice. (**a**,**b**,**d**,**e**) Representative actograms under 3.5/3.5-h LD cycle (**a** White light, **b** UVA light, **d** UVA light + Saline injection under the scalp, **e** UVA light + India ink injection under the scalp). (**c**) Masking ratio under white or UVA light. Mean ± SEM (n = 6; *p < 0.05, Student’s *t*-test). (**f**) Masking ratio under white or UVA light, with saline or India ink injection. Mean ± SEM (n = 5–7). (**g**,**h**) Temperature changes and representative actograms under UVA (**g**) or white (**h**) light–dark conditions.

Due to the phase-dependency of the onset of masking (Fig. 1g), we next exposed mice to an ultradian 7-hour (3.5/3.5-h) UVA-LD cycle^6,18^ (Supplementary Fig. S1a). Because mice cannot entrain their circadian rhythms to this 7-hour periodicity, the light and dark portions of the cycle move across the circadian cycle in this ultradian regime; thus, the pattern of activity under this regime represents masking effects rather than activity controlled by the circadian oscillator. Under this ultradian UVA-LD cycle, blinded mice confined their activity mostly to the dark phase but were active randomly under a white-LD cycle (Fig. 2a–c), consistent with the results shown in Fig. 1. Next, we tested whether this negative masking behavior was light-dependent by injecting India ink under the scalp^19^. This treatment reduced the intensity of light that penetrated the skull to approximately 1/200 of that in intact mice. However, India ink injection did not affect masking behavior (Fig. 2d–f), suggesting that mice were not using information from UVA light. When we examined more carefully the activity rhythms in Fig. 2g, we noticed a time lag between UVA light onset and activity offset. Based on this observation, we speculated that an ambient temperature (T_a_) rise induced by UVA light might be causing the masking behavior. Indeed, when we measured temporal changes in T_a_ inside the light-tight box, we observed a significant increase in T_a_ following UVA light exposure (Fig. 2g). No such temperature rise was observed under white-LD cycles (Fig. 2h). The concurrence between high T_a_ (>30 °C) and masking behavior suggested that the T_a_ change caused by the UVA light apparatus was triggering negative masking.

### T_a_ cycles induce negative masking behaviors in mice

**Figure 3:**
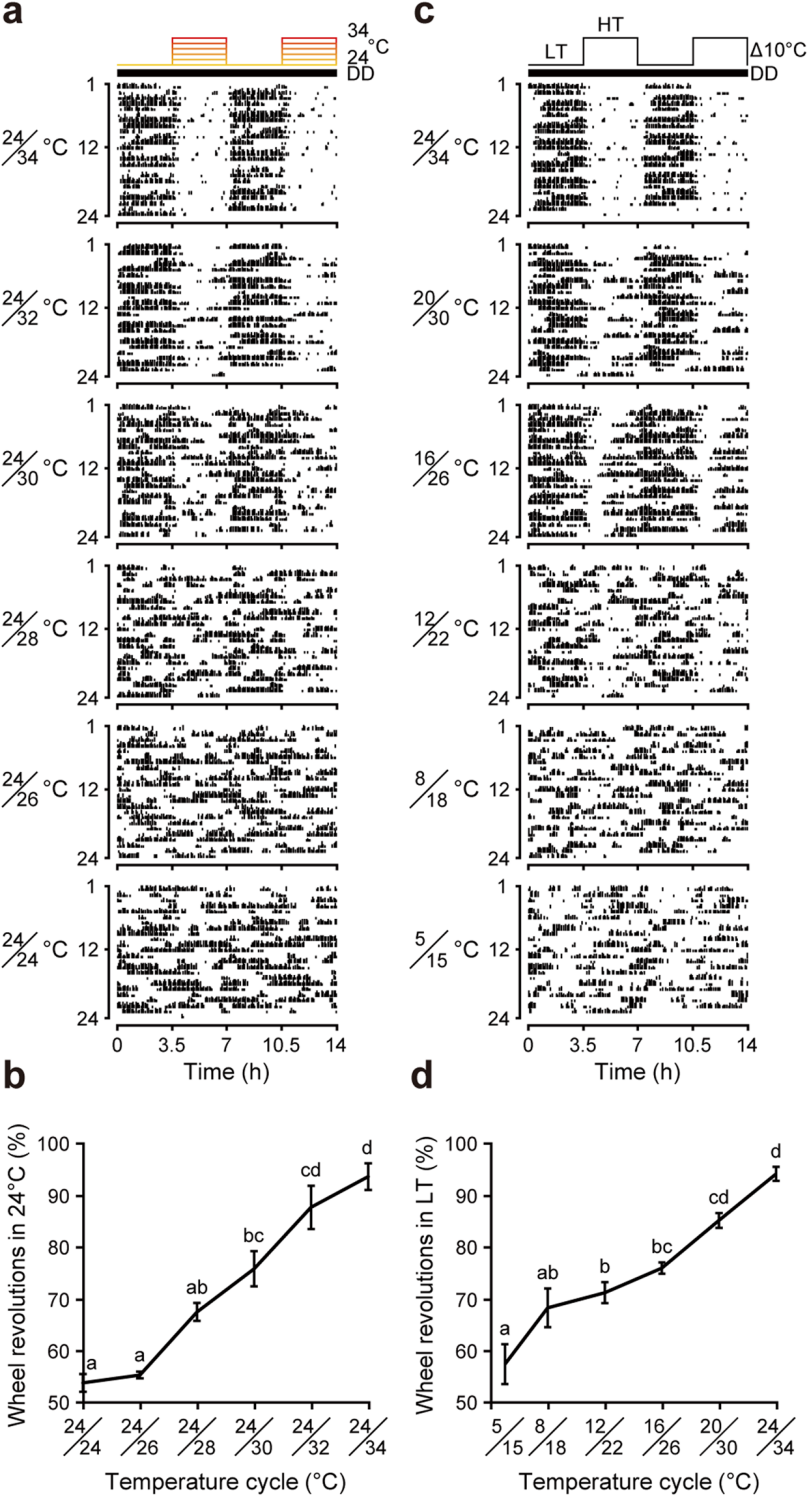
T_a_ cycles induce negative masking behaviors in mice. (**a**) Representative actograms of C57BL/6J mice. Locomotor activities were recorded for 1 week in DD under various 3.5/3.5-h T_a_ cycles. Temperature cycle patterns are illustrated at the top. (**b**) Masking ratio under each T_a_ cycle. Mean ± SEM (n = 4; *p* < 0.01, ANOVA, *F*_5, 23_ = 38.94; *p* < 0.01, Scheffé’s *post hoc* test). (**c**) Representative actograms of C57BL/6 J mice in DD under various 3.5/3.5-h T_a_ cycles in which the temperature difference was held constant at 10 °C. LT: low temperature; HT: high temperature. (**d**) Masking ratio under each 10 °C temperature difference cycle. Mean ± SEM (n = 8; *p* < 0.01, ANOVA, *F*_5, 47_ = 26.45; *p* < 0.01, Scheffé’s *post hoc* test). Different letters in (**b**,**d**) indicate significant differences between different groups.

To confirm that T_a_ cycles were indeed causing the masking behavior, we next examined the effect of various T_a_ cycles in intact C57BL/6 J mice under constant darkness (DD). In mice, the thermoneutral zone ranges from 26 °C to 34 °C^20,21^. When mice were exposed to 3.5/3.5-hour cycles of various temperature differences (24/24 °C, 24/26 °C, 24/28 °C, 24/30 °C, 24/32 °C, 24/34 °C; Supplementary Fig. S1b), negative masking was observed during exposure to the higher temperature, and the increase in the masking ratio was directly proportional to the increase in the temperature difference (Fig. 3a,b). We also analyzed wheel-running activity rhythms under various T_a_ cycles in which the difference between maximum and minimum temperature was held constant at 10 °C. Masking behavior was clearer in cycles at higher temperatures (Fig. 3c,d).

### Impaired negative masking behavior in *Trpm2* KO and *Trpm8* KO mice

**Figure 4:**
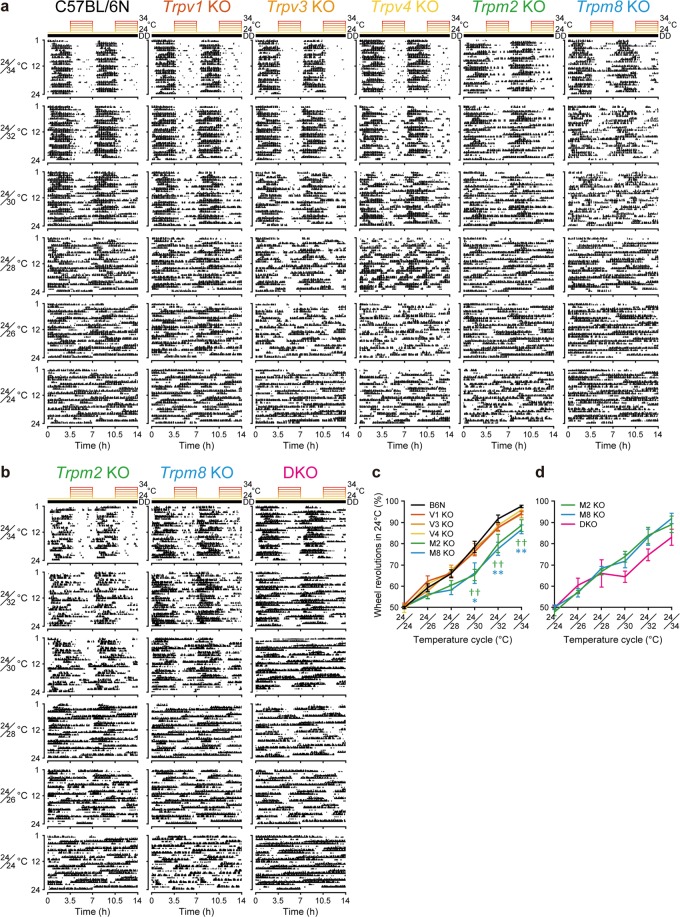
Impaired negative masking behaviors in *Trpm2* KO and *Trpm8* KO mice. (**a**) Representative actograms of C57BL/6 N (B6N), *Trpv1* KO, *Trpv3* KO, *Trpv4* KO, *Trpm2* KO, and *Trpm8* KO mice. (**b**) Representative actograms of *Trpm2* KO, *Trpm8* KO, and *Trpm2/8* DKO mice. (**c**) Masking ratio of each TRP KO mouse shown in (**a**). Mean ± SEM (n = 8–10 [B6N], 7–10 [V1], 6–8 [V3], 3–6 [V4], 5–8 [M2], 5–7 [M8]; ^††^*p* < 0.01, *a priori* Dunnett’s test [B6N vs. *Trpm2* KO]; **p < 0.01, *p < 0.05, *a priori* Dunnett’s test [B6N vs. *Trpm8* KO]). (**d**) Masking ratio of *Trpm2/8* DKO mice shown in (**b**). Mean ± SEM (n = 3–8 [M2], 3–7 [M8], 4–7 [DKO]).

To date, 10 TRP channels have been identified as thermosensors in mammals^22,23^ (Supplementary Fig. S2). TRPA1 and TRPM8 are cold-activated channels, whereas TRPV1, TRPV2, and TRPM3 are heat-activated. On the other hand, TRPV3, TRPV4, TRPM2, TRPM4, and TRPM5 are activated by warm temperatures^22,23^. The thermosensor(s) that mediate temperature-dependent masking behaviors are unknown. To identify these thermosensor(s), we examined masking in all available TRP channel–null mice (*Trpv1/3/4* and *Trpm2/8*) in our laboratory. Because the genetic background of these knockout mice was C57BL/6N, we used C57BL/6N mice as control animals. *Trpv1*-, *Trpv3*-, and *Trpv4*-null mice showed no differences in behavior compared to wild-type mice. However, *Trpm2*- and *Trpm8*-null mice exhibited impaired masking behaviors in response to T_a_ cycles (Fig. 4a,c). Furthermore, we bred *Trpm2*- and *Trpm8*-null mice to generate double-KO (DKO) mice. Although the observed masking ratio in DKO mice was not significantly different compared to single-KO mice, DKO mice tended to exhibit more severe phenotypes at higher-temperature cycles (e.g., 24/30 °C, 24/32 °C, and 24/34 °C) (Fig. 4b,d). When we compared the total activity of KO mice used in this study, statistically significant differences were only detected in *Trpv4*-null mice at 24/24 °C and 24/26 °C cycles (Supplementary Fig. S3). The low activity observed in *Trpv4*-null mice is likely due to muscular atrophy concomitant with hereditary neuropathies in this mutant^24^. Since we evaluate masking by calculating the activity ratio between different temperatures, the motor dysfunction observed in *Trpv4*-null mice does not affect our results.

### Possible neural circuit underlying negative masking behavior

**Figure 5:**
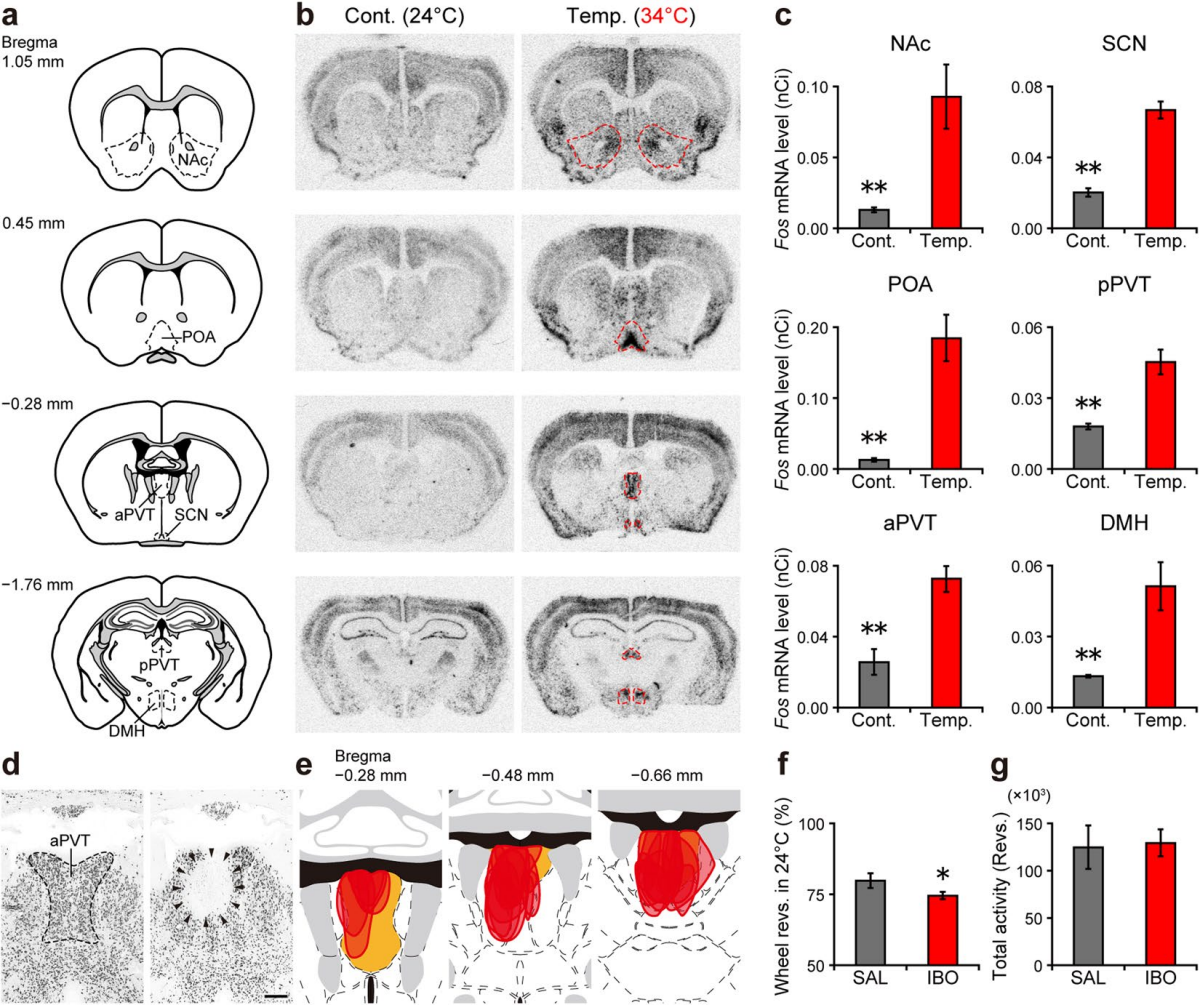
Possible involvement of the aPVT in negative masking behavior. (**a**) Schematic drawings of coronal mouse brain. Black dashed lines indicate the nuclei described in this study. Drawings in (**a**,**e**) were modified from the mouse brain atlas published by Allen Institute for Brain Science (©2004 Allen Institute for Brain Science. Allen Mouse Brain Atlas. Available from: http://mouse.brain-map.org). (**b**) Representative autoradiograms of *Fos* expression with/without temperature stimulus. Red dashed lines correspond to the black dashed lines in (**a**). (**c**) Densitometric quantifications of each nucleus. Mean ± SEM (n = 4; **p < 0.01, Student’s *t*-test). (**d**) NeuN immunohistochemistry in the aPVT of saline-injected control (left) and ibotenate-injected aPVT-lesioned (right) mice. Lesioned area is delineated by arrowheads. Scale bar: 200 µm. (**e**) Schematic drawings of aPVT areas lesioned by ibotenate injection (Bregma **−**0.28 to **−**0.66 mm). Orange areas indicate the aPVT. Lesioned areas are delineated and translucently filled in red. All lesioned areas and individual data are shown in Supplementary Figs S4 and S5, respectively. (**f**,**g**) Masking ratio (**f**) and total activity (**g**) of saline-injected control (left) and ibotenate-injected aPVT-lesioned (right) mice. Locomotor activity was recorded for 1 week in DD under a 3.5/3.5-h T_a_ cycle (24/30 °C). Mean ± SEM (n = 9 [saline], 18 [ibotenate]; *p < 0.05, Student’s *t*-test).

To identify the neural circuit that regulates temperature-dependent masking behavior, we first examined the expression of a histochemical marker of neuronal activation, *Fos*, during temperature-dependent masking behavior by *in situ* hybridization. Thirty minutes of masking-inducing warm temperature stimulus (34 °C) increased *Fos* expression in several nuclei, including the nucleus accumbens (NAc), preoptic area (POA) of the hypothalamus (mainly the median preoptic nucleus [MnPO]), anterior paraventricular nucleus of the thalamus (aPVT), SCN, posterior PVT (pPVT), and dorsomedial nucleus of the hypothalamus (DMH) (Fig. 5a–c). The neural connections among these nuclei are well characterized, and the PVT appears to be an important interface for the regulation of temperature-dependent negative masking behavior (see Discussion). To confirm this hypothesis, we performed chemical lesioning of the aPVT by injecting ibotenate (Fig. 5d–g, Supplementary Figs S4 and S5). Lesion of the entire aPVT was technically impossible due to the high mortality rate caused by repeated injections. However, partial lesions of the aPVT were possible and led to a small (approximately 7%), but significant decrease in temperature-dependent negative masking behavior compared to saline-injected control mice (Fig. 5d–f). Note that total activity did not differ between these two groups (Fig. 5g) and that lesions outside the aPVT had no effect on negative masking behavior (Supplementary Fig. S6). These results suggest that the aPVT is involved in regulation of negative masking behavior.

## Discussion

In previous studies, we found that UVA-sensitive OPN5-positive cerebrospinal fluid-contacting neurons within the hypothalamus are deep brain photoreceptors that regulate seasonal reproduction in birds^8,9^. Since light penetrates into the brain of small mammals^10^ and OPN5 is reportedly expressed in the mammalian brain^11,12^, we first examined whether mice have the capacity for extra-ocular photoreception using blinded mice. Although we observed clear suppression of locomotor activity by UVA light exposure (350–400 nm), this behavior was induced by the temperature rise caused by the UVA light source, rather than by UVA light itself (Figs 1 and 2). We therefore conclude that mice do not have the capacity for extra-ocular photoreception as previously suggested^14,15^.

Our knowledge of the regulatory mechanisms responsible for masking behavior is significantly less than our understanding of circadian photoentrainment, despite the fact that both phenomena are important for determining the temporal distribution of locomotor activity (i.e., temporal niche). In this study, we observed negative masking-like behavior (i.e., acute suppression of locomotor activity) in mice at higher temperatures, consistent with a previous report^25^ (Figs 1–3). When activity was plotted on a 24-h time scale (Supplementary Figs S7 and S8), suppression of locomotor activity was only observed when mice were exposed to the higher temperatures. Importantly, these double-plotted actograms showed that mice were free-running during 3.5/3.5-h T_a_ cycles (Supplementary Fig. S7) and continued to free-run after transfer from T_a_ cycles to constant conditions (constant 24 °C with DD) (Supplementary Fig. S8). Thus, the observed suppression of locomotor activity at higher temperatures is clearly negative masking behavior rather than entrainment. In the present study, we evaluated masking behavior by measuring wheel-running activity. One could speculate that animals might reduce their wheel-running activity to prevent hyperthermia at high ambient temperatures and that negative masking behavior depends on the intensity of physical activity. We therefore analyzed total activity during masking behavior (Supplementary Fig. S9). Total activity under 24/34 °C tended to be lower than that of 24/24 °C cycles, but there was no significant difference (Supplementary Fig. S9a). By contrast, total activity under 24/34 °C was higher than that at 5/15 °C (Supplementary Fig. S9b). Therefore, we believe that temperature-dependent masking behavior does not necessarily depend on the intensity of the physical activity.

Although several TRPA channels (e.g., dTRPA1 and Pyrexia) are involved in the regulation of activity levels during the afternoon, rhythmicity of temperature preference, and temperature synchronization of the circadian clock in *Drosophila*^26,27^, the thermosensors regulating behavioral rhythms in vertebrates remain unknown. Multiple TRP channels covering a wide range of temperatures have been identified in mammals^22,23^ (Supplementary Fig. S2). By analyzing all the available TRP channel–knockout mice, we discovered impaired negative masking behaviors in *Trpm2*- and *Trpm8*-null mice (Fig. 4). TRPM2 is a warm-sensitive thermosensor that is activated within the physiological range of body temperature and is involved in the sensation of environmental warmth^28^, reduction of fever size by detecting hyperthermic temperature in the POA^29^, fever-associated enhancement of macrophage phagocytosis^30^, and body temperature–evoked insulin secretion^31^. On the other hand, TRPM8 is a cold-sensitive thermosensor that also acts as a menthol receptor^32,33^. TRPM8 deficiency leads to impairment in sensing unpleasant cold stimuli, including cold-inducing icilin application and acetone cooling^34–36^. Importantly, these two TRP channels are intimately involved in thermoregulation^29,37–39^. Our results demonstrate that warm-sensitive TRPM2 and cold-sensitive TRPM8 also act as thermosensors for the regulation of temperature-dependent negative masking behavior. This seems plausible because to sense absolute temperature value, at least two thermosensors that span different temperature ranges (e.g., cold-sensitive and warm-sensitive channels) are required^40^. However, we do not fully comprehend why both *Trpm2*- and *Trpm8*-null mice showed the same masking behavior within the exact same temperature range. Although *Trpm2*/*Trpm8* DKO mice tend to be more severely impaired than the single-KO mice (Fig. 4b,d), temperature-dependent masking behavior is not abolished. These results are consistent with the fact that individual TRP channel knockout models, and even DKO mice, often do not display strong temperature phenotypes^41^. This is because many TRP channels detect overlapping temperatures, and extensive compensation occurs among redundant temperature detectors. In any case, our data suggests the involvement of additional thermosensor(s) in negative masking behavior. Clearly, further investigation is required to identify these remaining thermosensor(s). Since knockout mice for warm sensitive TRPM4 and TRPM5 were unavailable, they are obvious potential candidates.

**Figure 6:**
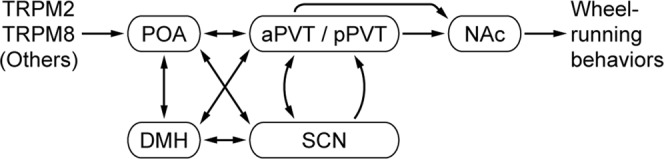
Possible neural circuit regulating temperature-dependent negative masking behavior in mice. Temperature information detected by thermosensors (e.g., TRPM2 and TRPM8) is sent to the thermoregulatory center, the POA, which sends direct and indirect (via DMH and/or SCN) projections to the aPVT/pPVT. The aPVT/pPVT and SCN are reciprocally connected. The aPVT/pPVT sends projections to the NAc, which is involved in the regulation of wheel-running behaviors. Arrows between nuclei indicate direct connections^5,48–56^. Note that TRPM2 is also expressed in the POA^29^.

Some photoreceptors (i.e., melanopsin [Opn4]-expressing retinal ganglion cells, rods and cones)^6,42–44^ and several brain regions (i.e., IGL and olivary pretectal nucleus [OPN])^45,46^ are thought to mediate light-dependent masking behavior; however, the brain regions and neural circuits that mediate temperature-dependent masking behavior remain completely unknown. Expression analysis of the neuronal activation marker, *Fos*, reveals that several brain regions (NAc, POA, aPVT, SCN, pPVT, DMH) are activated by an acute increase in T_a_ that induces negative masking behavior (Fig. 5a–c). The neural connections between these nuclei are well characterized. Environmental temperature detected by thermosensors located in the skin and the brain is transmitted to the POA, the mammalian thermoregulatory center^47^. The POA, PVT, DMH, and SCN are mutually connected^5,48–55^. Direct^49–52^ and indirect projections through the DMH^48–52^ or the SCN^5,49–55^ connect the POA to the PVT. The PVT is reciprocally connected with the SCN; aPVT neurons send projections to the SCN, and SCN neurons project to the aPVT and pPVT^5,49–55^. The aPVT and pPVT send dense projections to the NAc^50–53,56^, an area of the striatum that acts as a limbic–motor interface to mediate a variety of behaviors, including motivation, locomotion, reward, and wheel-running activity^57–59^. Notably, in this regard, wheel-running is considered a reward to rodents^60^. Thus, the PVT appeared to be an important interface for the regulation of temperature-dependent negative masking behavior. Interestingly, we observed a slight decrease in masking behavior by partial lesion of the aPVT, suggesting the possible involvement of this nucleus in the regulation of negative masking behavior (Fig. 5d–f). Based on these findings, we propose a neural circuit responsible for regulating temperature-dependent negative masking behavior in mice (Fig. 6).

Recent studies have shown that the behavior of animals differs markedly between laboratory conditions (rectangular light/dark cycles and constant warm temperature) and natural conditions (gradually changing light intensity and temperature)^61–66^. Entrainment and masking are two independent processes that determine the timing of activity (temporal niche). Although a great deal of effort has been devoted to understanding the mechanisms underlying photoentrainment, the mechanisms responsible for masking behavior have remained unknown. We report here that two thermosensors, TRPM2 and TRPM8, are involved in the regulation of temperature-dependent negative masking behavior. Moreover, the PVT is likely to be an important interface for this adaptive behavior. We believe that our findings will contribute to a greater understanding of masking behavior, and eventually, to the regulatory mechanisms involved in temporal niche switching (e.g., diurnality and nocturnality).

## Methods

### Animals

C57BL/6J and C57BL/6N mice were purchased from a local dealer (Japan SLC, Inc.). TRP channel KO mice (*Trpv1*^67^, *Trpv3*^68^, *Trpv4*^69^, *Trpm2*^70^, *Trpm8*^35^) backcrossed more than 5 times with C57BL/6N mice were used in this study. *Trpm2/Trpm8* DKO mice (F2 progeny) generated by intercrosses between *Trpm2* KO mice and *Trpm8* KO mice were also used in this study. We used male mice whenever possible. If sufficient numbers of males were not available, we used female mice: *Trpv1* KO (4 females out of 10) and *Trpm8* KO (3 females out of 7) mice in Fig. 4c; *Trpm2* KO (5 females out of 8), *Trpm8* KO (5 females out of 7) and *Trpm2*/*Trpm8* DKO (5 females out of 7) mice in Fig. 4d. The total number of male and female mice was too small for a proper statistical comparison, and further detailed analyses are required to confirm any sex differences. However, of the mice used in this study, no clear differences were observed between the sexes. All animals were housed in a controlled environment (white-LD cycle [12/12-h]; room temperature 22–24 °C) prior to experiments. Food and water were provided *ad libitum*. All animal procedures in this study were approved by the Animal Experiment Committee of Nagoya University, and all experiments were performed in accordance with the relevant guidelines and regulations.

### Effect of UVA light exposure on wheel-running activity of blinded mice

Eight-week-old male C57BL/6J mice were kept in individual cages (14.8 × 25.0 × 14.8 cm) equipped with running wheels (10.0 cm diameter), and the cages were placed together in a light-tight box (136.7 × 42.5 × 42.5 cm). Light in the box was provided by fluorescent lamps (white light: FHF32EX-N-H, Panasonic, 4,150 lux at the top of the cage; UVA light: TL-D 36 W/08 low-pressure mercury vapor fluorescent lamp, Philips, peak wavelength 365 nm with half-bandwidth 13.9 nm). Both eyes were surgically removed (enucleated) under isoflurane anesthesia (Ex group). Two weeks after the surgery, the light source was changed to UVA light (light intensity ~15.3 log photons cm^−2^ s^−1^, less than the intensity under direct sunlight in Nagoya, Japan). Wheel-running activities were continuously recorded using the Chronobiology Kit (Stanford Software Systems). T_a_ in the light-tight box was measured using temperature data loggers (Thermochron type-G, KN Laboratories), and data were retrieved using the ThermoManager software (KN Laboratories).

### Evaluation of masking behavior

A 3.5/3.5-h LD cycle and a T_a_ cycle were used to quantitatively evaluate masking behavior (Supplementary Fig. S1). The number of wheel revolutions in the dark- or lower temperature-phase compared to the total number of revolutions was defined as the masking ratio. When the animal’s activity is unaffected by environmental stimuli, the masking ratio is close to 50%. A Biomulti incubator (LP-30CCFL-8CTAR, Nippon Medical & Chemical Instruments) was used for temperature control. Using this equipment, we could control the T_a_ and light conditions independently. T_a_ cycles were examined in the following order: 24/28 °C, 24/30 °C, 24/32 °C, 24/34 °C, 24/24 °C, 24/26 °C.

### India ink injection under the scalp

India ink (Tenboku, Kuretake) was autoclaved the day before injection. Autoclaved India ink (300 µl) was injected between the scalp and the skull of mice using a 1-ml syringe and 26 G needle under isoflurane anesthesia. The same amount of saline was injected into the control group.

### *In situ* hybridization of *Fos* mRNA

Eight-week-old male C57BL/6N mice were placed in individual cages equipped with running wheels and were entrained to a white-LD cycle (12/12-h) for 2 weeks. During this time, the T_a_ was maintained at 24 °C. Subsequently, a 30-min warm-temperature stimulus (34 °C) was given 4-hour after the light offset (Zeitgeber time [ZT] 16) in the temperature stimulus group (Fig. 5b-right). In the control group, T_a_ was held constant at 24 °C (Fig. 5b-left). Because *Fos* mRNA expression peaks 30 minutes after stimulation^71^, brains were collected using a pair of night-vision goggles (Ninox, Armasight) and rapidly frozen in dry ice at ZT16.5. Non-perfused frozen sections (20-µm thickness) were prepared using a cryostat (CM3050 S, Leica Microsystems) and examined with ^33^P-labeled oligonucleotide probes. Four 45-mer oligonucleotide probes were designed against the mouse *Fos* gene (GenBank: NM_010234) and used as a mixture to increase the sensitivity. Hybridization was carried out overnight at 42 °C. Two high-stringency post-hybridization washes were performed at 55 °C. Sections were air-dried and exposed to BioMax MR Film (Eastman Kodak) for 4 weeks with ^14^C-Standard slide (American Radiolabeled Chemicals). Densitometric quantification of hybridization signals was performed using the Multi Gauge software (Fujifilm). The probe sequences were as follows:

5′-tcactgctcgttcgcggaaccgccggctctatccagtcttctcag-3′

5′-tccagggaggccacagacatctcctctgggaagccaaggtcatcg-3′

5′-atctggcacagagcgggaggtctctgagccactgggcctagatga-3′

5′-ctggaggccagatgtggatgcttgcaagtccttgaggcccacagc-3′

### Ibotenate injection into the aPVT

Ibotenate injection was performed in accordance with the earlier study^72^. Mice were deeply anesthetized with chloral hydrate (280 mg kg^−1^, i.p. injection with 7% solution). Subsequently, 5 mM ibotenate or saline (20–50 nl) was injected to the aPVT (coordinates: 0.2 mm caudal to bregma, 0.0 mm lateral to the midline, and 3.6 mm ventral to the skull surface). Due to the high mortality rate, each mouse received only one injection. Lesioned areas were evaluated by NeuN immunohistochemistry immediately after evaluation of masking behavior, and are depicted in Fig. 5e, Supplementary Figs S4 and S5. All behavioral analyses of ibotenate- or saline-injected mice were performed after >1 week of recovery.

### Immunohistochemistry

Immunohistochemistry was performed using rabbit monoclonal anti-NeuN antibody (ab177487, Abcam) (dilution 1:500) and N-Histofine Simple Stain Mouse MAX PO (R) (Nichirei Biosciences) with a standard protocol^73^.

### Statistical analysis

All data are shown as the mean ± SEM. Statistical analyses were performed using the Rayleigh test, Student’s *t*-test, or one-way ANOVA, followed by Scheffé’s *post hoc* test or *a priori* Dunnett’s test.

## Supplementary information

Supplementary information

## Data Availability

Any related data and/or information of this study are available from the corresponding author upon request.
