# Supplementary material for: Involvement of TRPM2 and TRPM8 in temperature-dependent masking behavior
Source: Sci Rep. 2019 Mar 6;9:3706. doi: 10.1038/s41598-019-40067-x (PMC6403366; doi:10.1038/s41598-019-40067-x)
Supplement: Supplementary file 1 — Supplementary information [file 41598_2019_40067_MOESM1_ESM.pdf]

Involvement of TRPM2 and TRPM8 in temperature-dependent masking behavior

Wataru Ota, Yusuke Nakane, Makiko Kashio, Yoshiro Suzuki, Kazuhiro Nakamura, Yasuo Mori, Makoto Tominaga, and Takashi Yoshimura

Supplementary Information

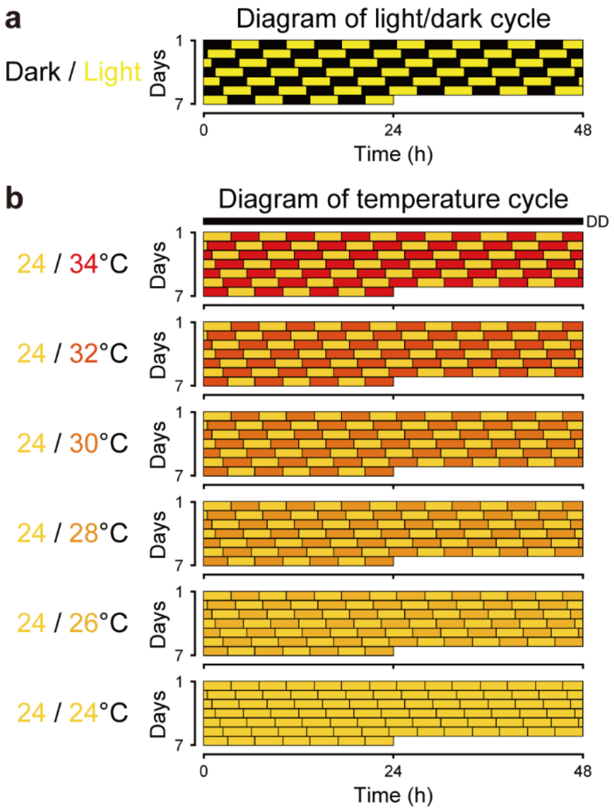

Supplementary Figure S1. Diagrams of 3.5/3.5-h cycles. (**a**) Diagrams of 3.5/3.5-h light/dark cycles used in Fig. 2. Dark and light phases are shown as black and yellow rectangles, respectively. (**b**) Diagrams of 3.5/3.5-h temperature cycles under constant darkness (DD) used in Figs. 3, 4, and 5f. Base temperature phases (24°C) are shown as yellow rectangles, and temperature stimulus phases are shown as rectangles of graded color (orange to red).

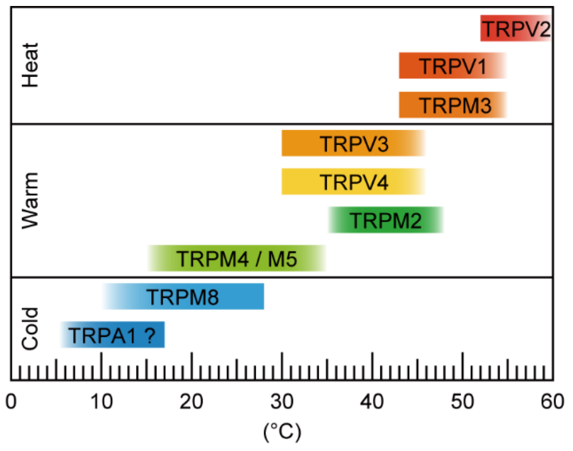

Supplementary Figure S2. Diagram of activation temperature ranges of TRP channels in mammals. Modified from Kashio and Tominaga[1,2].

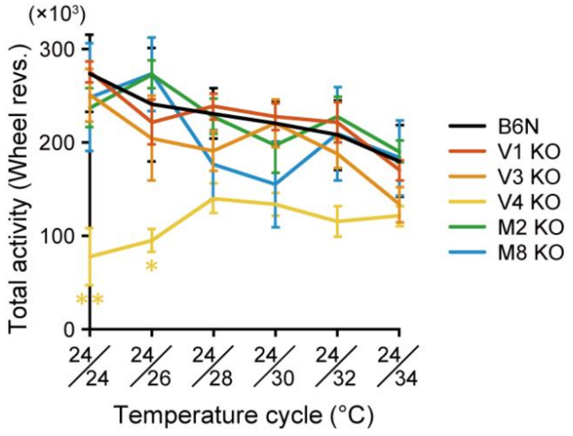

Supplementary Figure S3. Total activity of KO mice used in this study under different T_a cycles (24/24°C–24/34°C). Mean ± SEM (n = 8–10 [B6N], 7–10 [V1], 6–8 [V3], 3–6 [V4], 5–8 [M2], 5–7 [M8]; $^{**}p < 0.01$, $^{*}p < 0.05$, *a priori* Dunnett's test [B6N vs. *Trpv4* KO]).

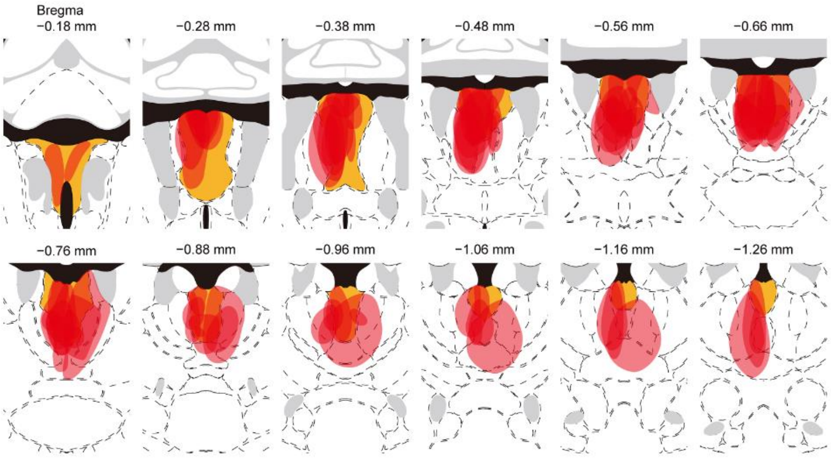

Supplementary Figure S4. Schematic drawings of aPVT areas lesioned by ibotenate injection (Bregma −0.18 to −1.26 mm). Orange areas indicate the aPVT. Lesioned areas from all animals (n = 18) are delineated and translucently filled in red. Drawings in this figure were modified from the mouse brain atlas published by Allen Institute for Brain Science (©2004 Allen Institute for Brain Science. Allen Mouse Brain Atlas. Available from: http://mouse.brain-map.org).

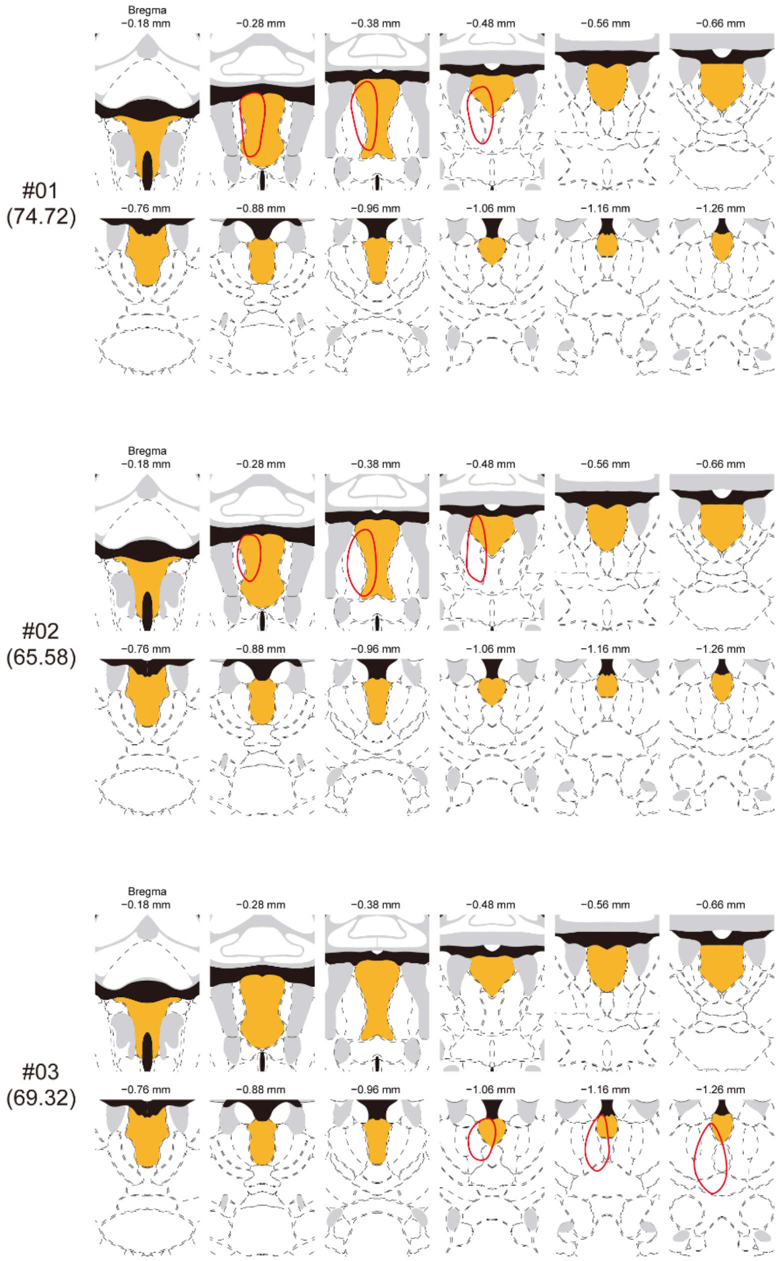

Supplementary Figure S5. Schematic drawings of aPVT areas lesioned by ibotenate injection (all individual data, n = 18; Bregma −0.18 to −1.26 mm). Individual numbers and their masking ratios are written on the left. Orange areas indicate the aPVT. Lesioned areas in each mouse are delineated by red lines. Drawings in this figure were modified from the mouse brain atlas published by Allen Institute for Brain Science (©2004 Allen Institute for Brain Science. Allen Mouse Brain Atlas. Available from: http://mouse.brain-map.org).

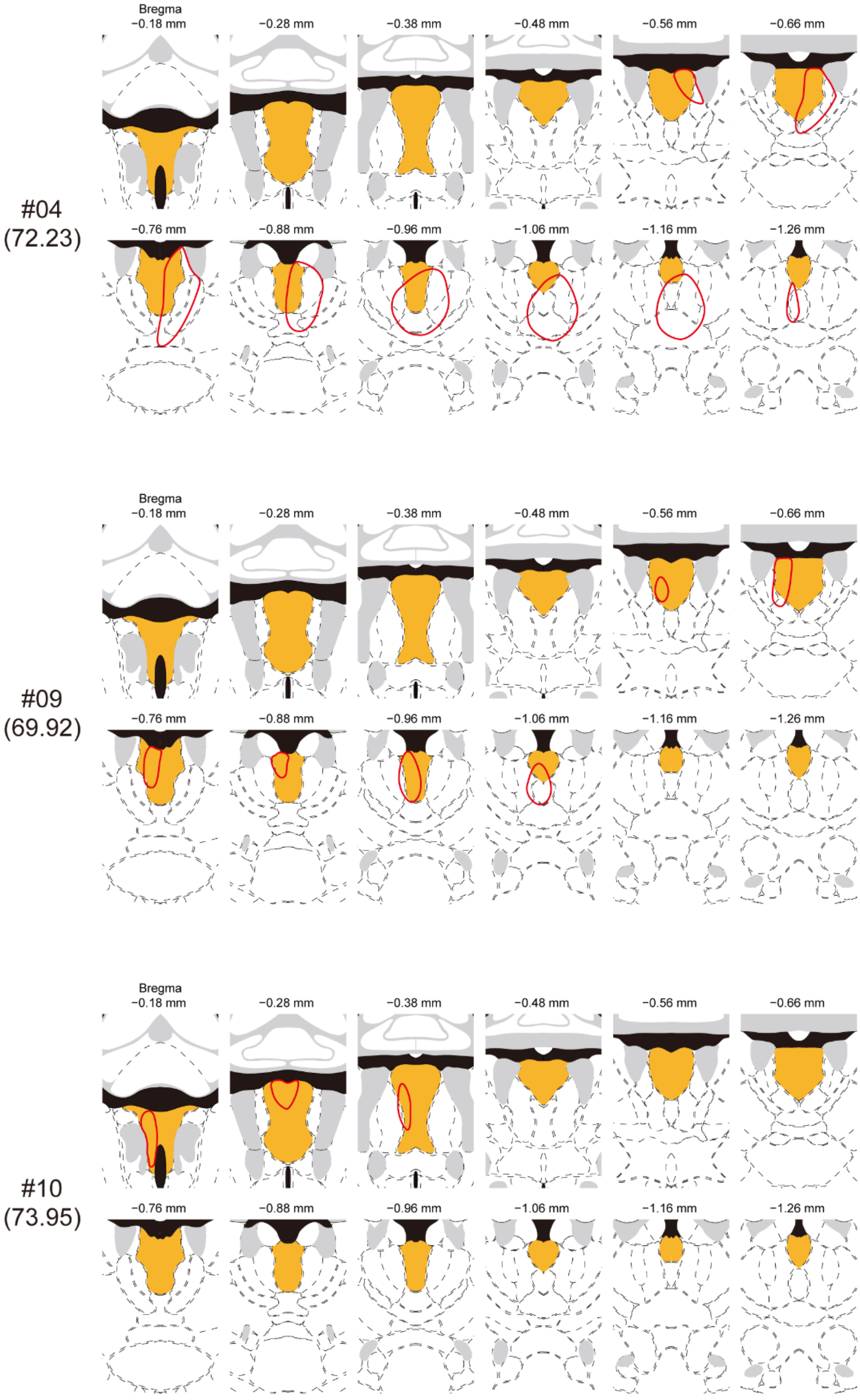

Supplementary Figure S5 (continued).

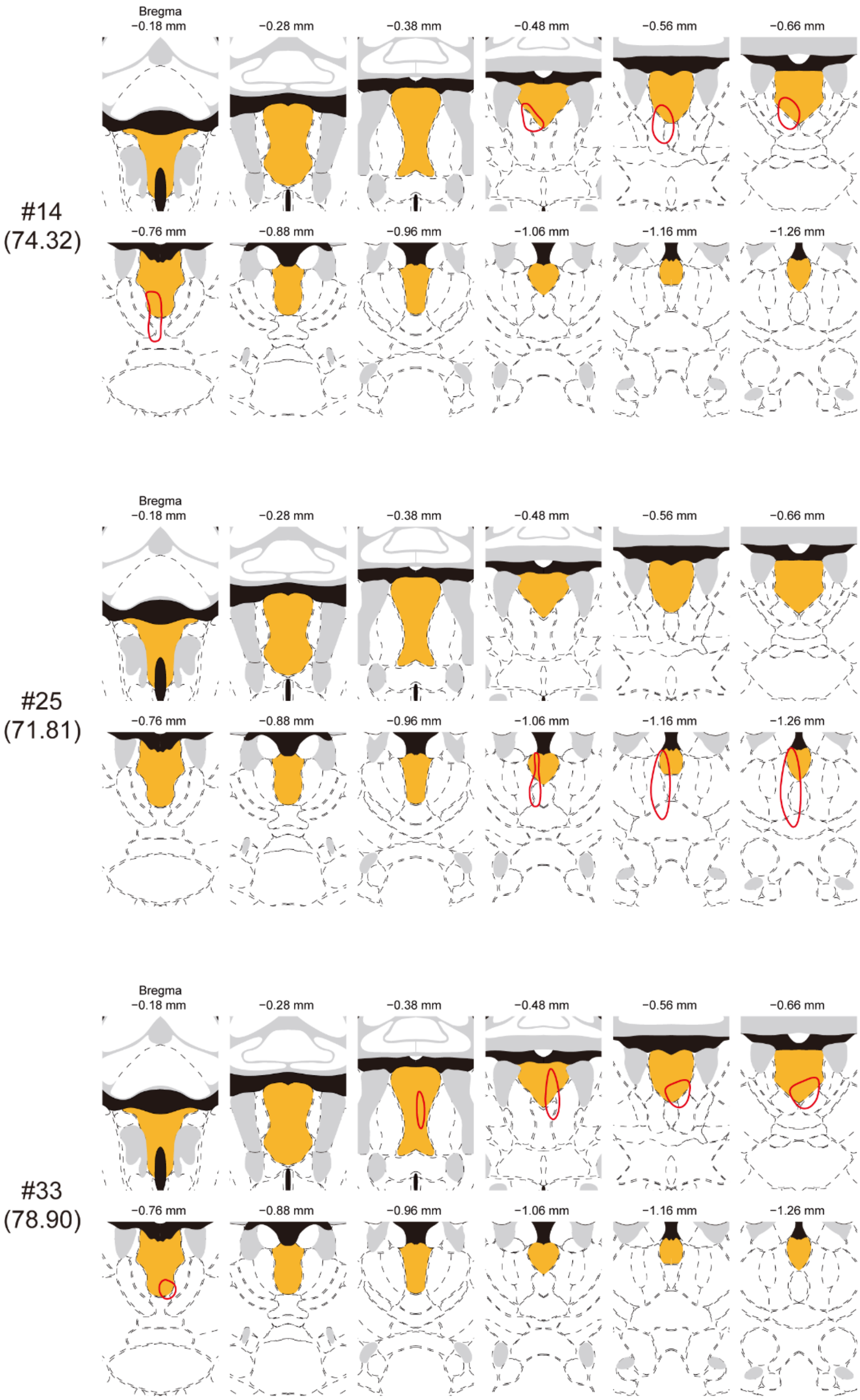

Supplementary Figure S5 (continued).

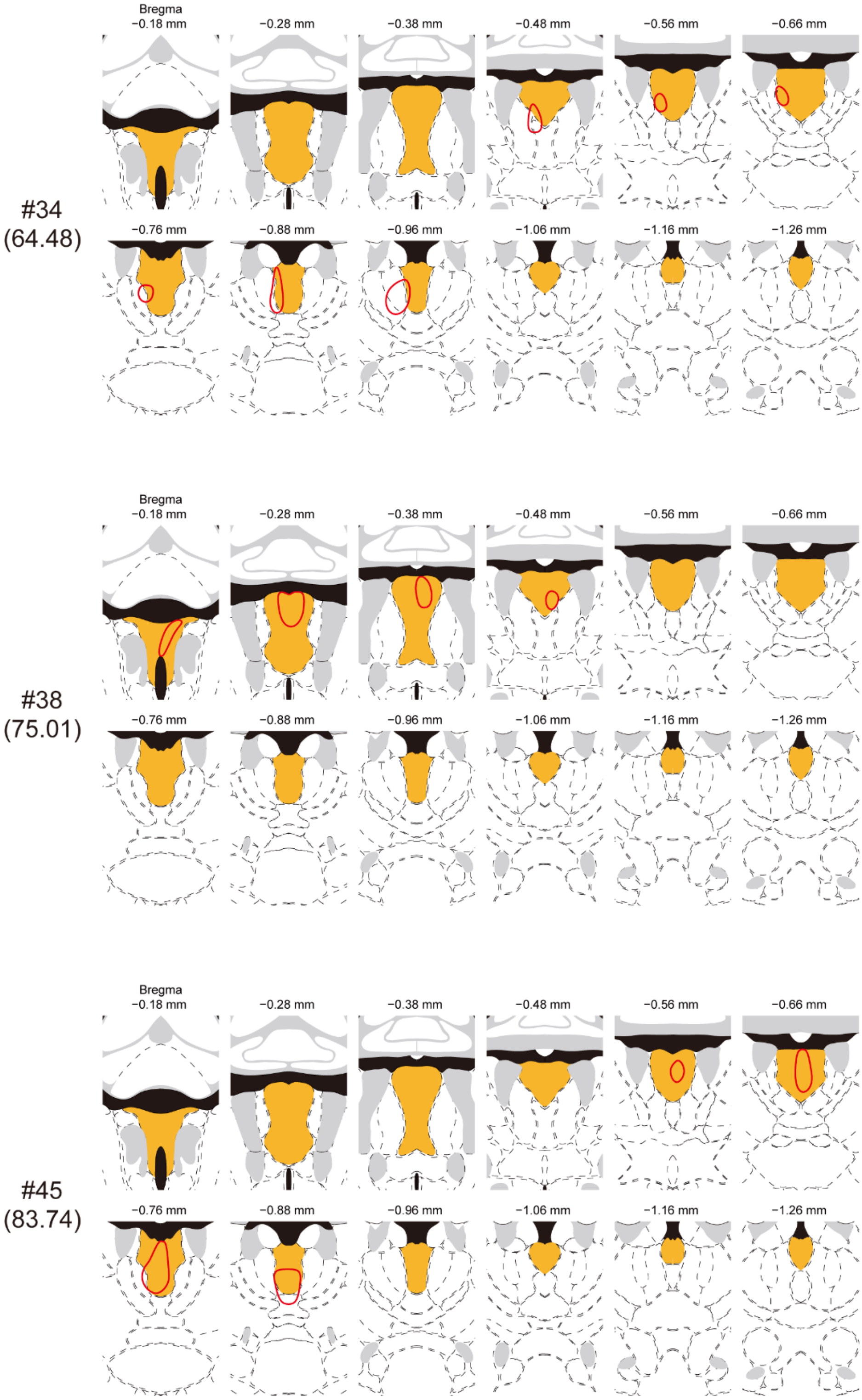

Supplementary Figure S5 (continued).

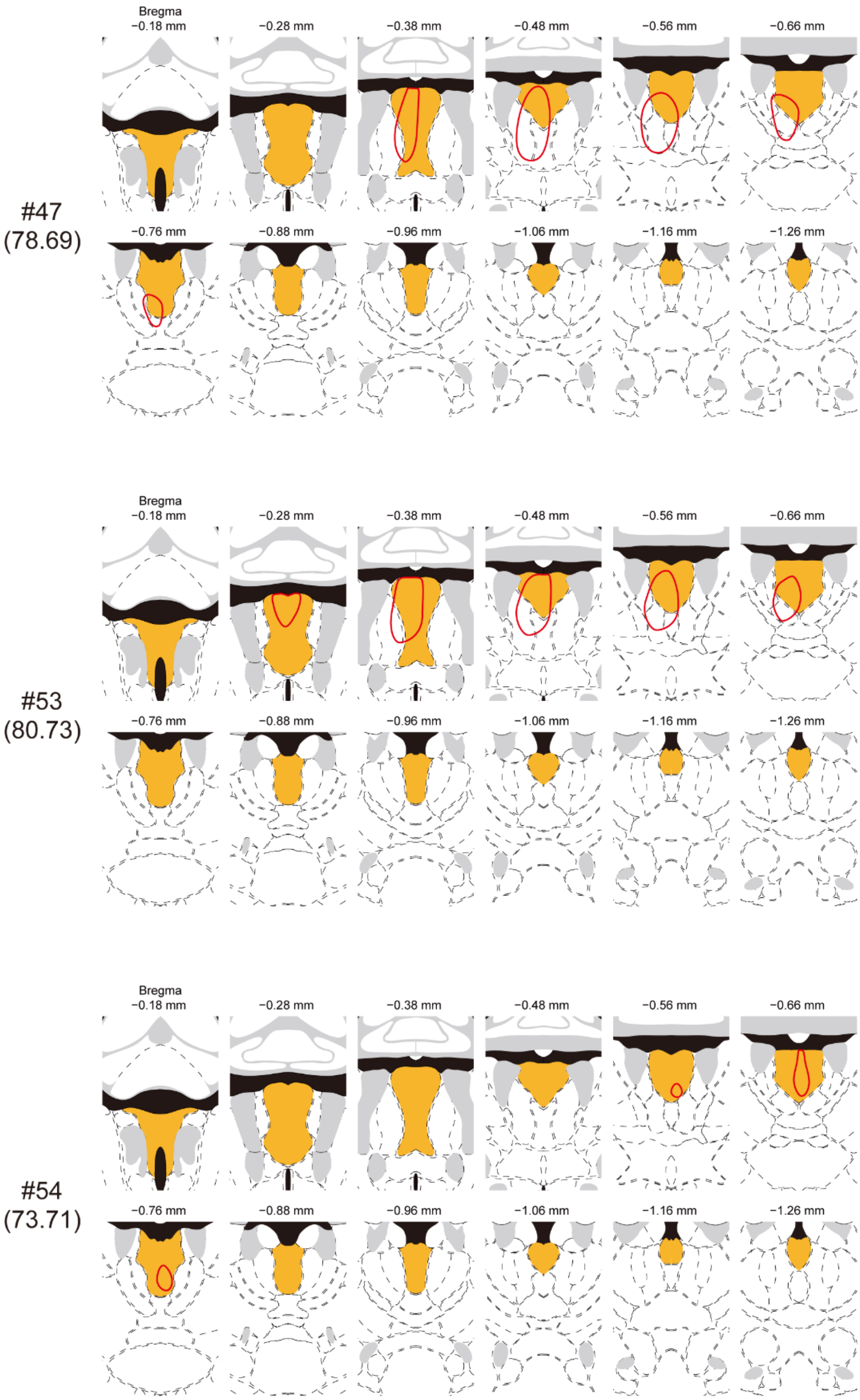

Supplementary Figure S5 (continued).

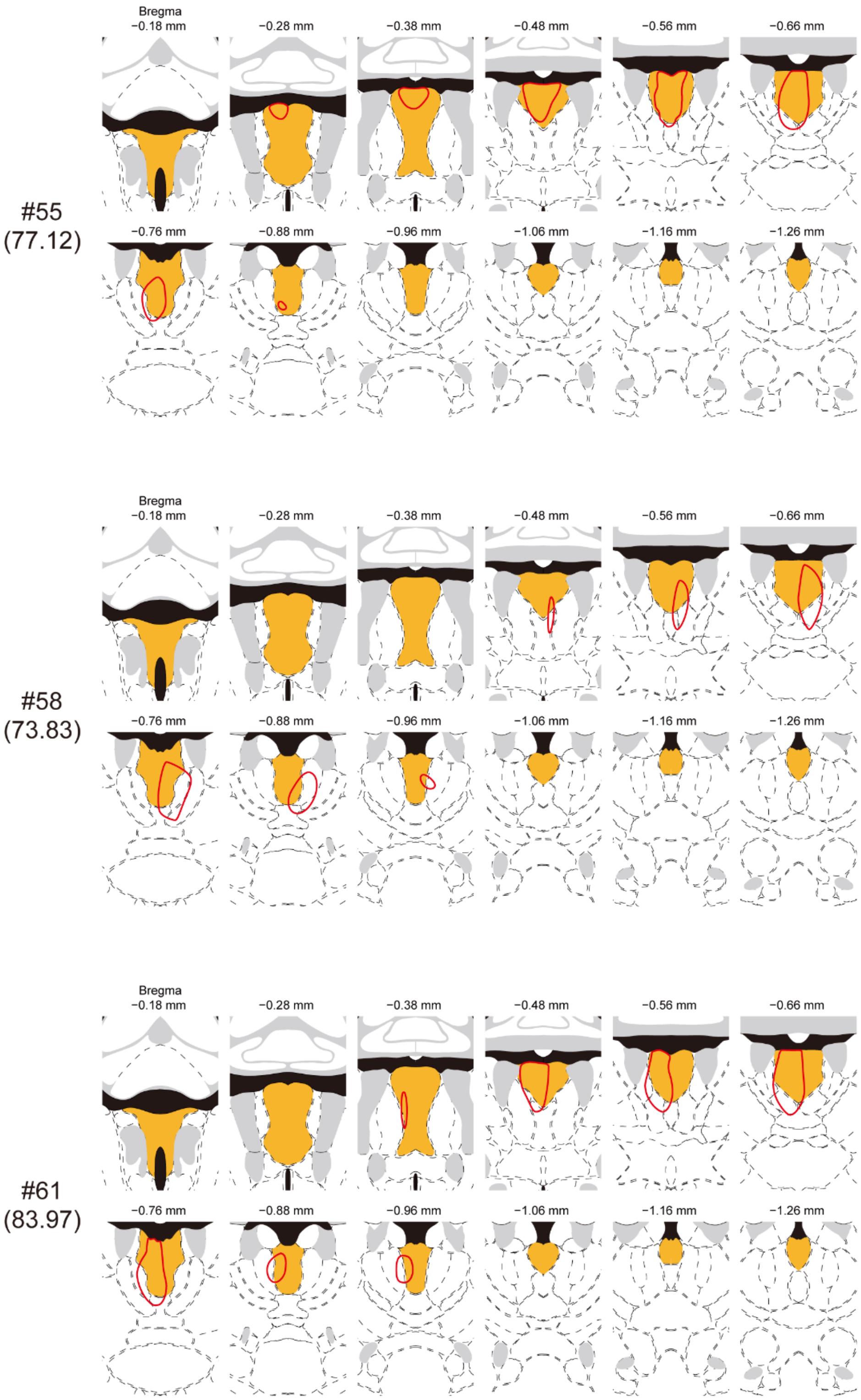

Supplementary Figure S5 (continued).

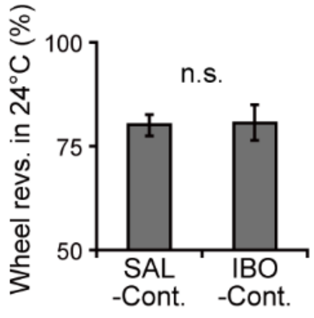

Supplementary Figure S6. Masking ratio of saline-injected control (left) and ibotenate-injected control outside the aPVT (right). Locomotor activities were recorded for 1 week in DD under a 3.5/3.5-h T_a cycle (24/30°C). Mean ± SEM (n = 9 [saline-injected control], 4 [ibotenate-injected control]; p > 0.05, Student's t-test).

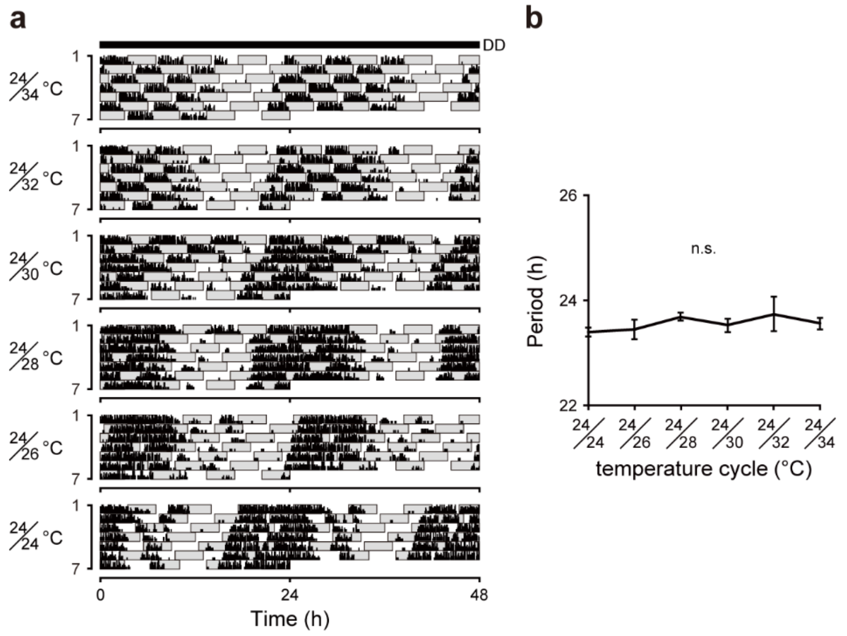

Supplementary Figure S7. Free-running rhythms of mice kept under various T_a cycles. (**a**) Representative actograms of C57BL/6N mice in DD under various T_a cycles. (**b**) Free-running periods under each T_a cycle. Mean ± SEM (n = 5–7; p > 0.05, ANOVA, $F_{5, 36}$ = 0.77; p > 0.05, Scheffé's post hoc test).

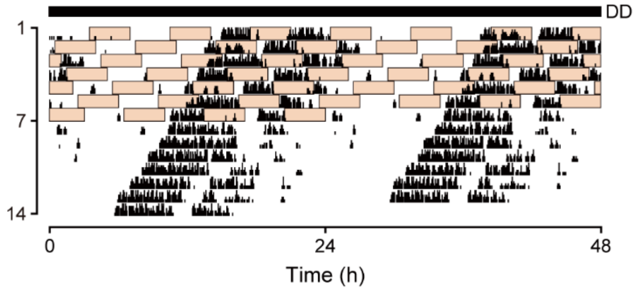

Supplementary Figure S8. Mice continue to free-run after transfer from T$_a$ cycles to constant conditions. Representative free-running activity of C57BL/6N mice during and after a masking-inducible T$_a$ cycle (24/34°C). Mice were kept in DD. Base temperature phases (24°C) are shown as a white background, and temperature stimulus phases (34°C) are shown as colored rectangles.

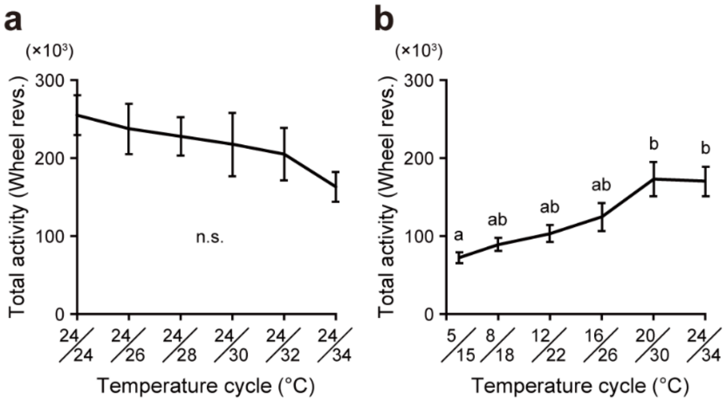

Supplementary Figure S9. Total activity of C57BL/6N mice under different T$_a$ cycles used in this study. (**a**) Total activity under different T$_a$ cycles (24/24°C–24/34°C). Mean ± SEM (n = 8; $p > 0.05$, ANOVA, $F_{5, 40} = 0.93$; $p > 0.05$, Scheffé's post hoc test). (**b**) Total activity under various T$_a$ cycles with a 10°C difference (5/15°C–24/34°C). Mean ± SEM (n = 8; $p < 0.01$, ANOVA, $F_{5, 46} = 7.43$; $p < 0.01$, Scheffé's post hoc test). Different letters indicate significant differences between different groups.

Supplementary References

1. Kashio, M. & Tominaga, M. Thermo-sensitive TRP channel sensing body temperature. in *Experimental Medicine* 512–518 (Yodosha, 2014).
2. Kashio, M. & Tominaga, M. The TRPM2 channel: a thermo-sensitive metabolic sensor. *Channels* **11,** 426–433 (2017).
